# Diffusion Bonding of Al_2_O_3_ Dispersion-Strengthened 316L Composite by Gleeble 3800

**DOI:** 10.3390/ma17102300

**Published:** 2024-05-13

**Authors:** Tétény Baross, Haroune Ben Zine Rachid, Péter Bereczki, Miklós Palánkai, Katalin Balázsi, Csaba Balázsi, Gábor Veres

**Affiliations:** 1 HUN-REN Centre for Energy Research, Konkoly-Thege M. St. 29-33, 1121 Budapest, Hungary; haroune.benzine@ek.hun-ren.hu (H.B.Z.R.); palankai.miklos@ek.hun-ren.hu (M.P.); balazsi.katalin@ek.hun-ren.hu (K.B.); veres.gabor@ek.hun-ren.hu (G.V.); 2 Faculty of Sciences and Technology, Mohamed Khider University, BP 145 RP, Biskra 07000, Algeria; 3 Department of Material Science, University of Dunaújváros, Táncsics M. Str. 1/A, 2400 Dunaújváros, Hungary; bereczkip@uniduna.hu; 4 Institute of Nuclear Technics, Budapest University of Technology and Economics, Műegyetem rkp. 3., 1111 Budapest, Hungary

**Keywords:** diffusion bonding, CDS composite, SPS, Gleeble

## Abstract

The aim of this work is to investigate the bonding properties of the ceramic dispersion-strengthened 316L (CDS-316L) composites with the reference 316L stainless steel (REF-316L) using a Gleeble 3800 physical simulator. In previous works, two different composites, REF-316L and 316L, with 1 wt% Al_2_O_3_ composite (CDS-316L) have been prepared by spark plasma sintering (SPS). In the present work, these specimens were diffusion-bonded using the following parameters: a temperature range of 950–1000 °C and a uniaxial pressure of 20–30 MPa. It was observed that the deformation of the CDS-316L during the uniaxial bonding process was higher compared to the 316L steel rods. The addition of alumina particles increased the micro-hardness of the 316L steel. The samples were broken in the CDS-316L zones, not at the diffusion-bonded interfaces. No diffusion zones have been observed within the investigated magnification for all composites, where the interfaces between the different specimens were well defined.

## 1. Introduction

Future fusion power plants shall be an alternative option for future energy production, with the essential aim of increasing the efficiency by increasing the operational temperature. The 316L ceramic dispersion-strengthened (CDS) steels appear as promising materials for application in future fusion power plants, with their good mechanical properties at elevated temperatures [1,2,3]. Presently, there is an increasing need for novel materials that can withstand the extreme working conditions of future generations of fusion and fission nuclear reactors [4,5], and this is of high importance since any material failure can result in a severe accident [6]. The advances in nanocomposite processing and manufacturing techniques for the materials are crucial and have a direct impact on their final properties. In a later fusion power plant in vacuum conditions, the plasma-facing first wall structures have to withstand extremely high heat loads, where good thermal contacts will be essential. There is no final design yet for the first wall of a next-generation fusion power plant, but based on ITER designs, diffusion bonding will be one of the key technologies for bonding flat surfaces, where bonded surfaces may serve as vacuum boundaries around cooling channels as well. Since the ODS (oxide dispersion strengthened) or CDS materials during a standard welding technique would melt, the clustering of oxide particles in the liquid phase would cause a strength reduction along the bond interface [7]. Following these, the present study investigates the diffusion bonding capability of a newly developed CDS material to standard steel. In earlier studies, we can see investigations for ODS steels compared to non-ODS reduced activation ferritic/martensitic (RAFM) steels [8] as well. Various types of solid-state diffusion bonding take place at the first walls of the fusion reactor for a good mechanical and thermal contact [2,3,9,10].

The traditional sintering processes involved in powder technology are known for their limitations in retaining nanosized particles in the final product [11,12]. On the other hand, powder metallurgy is a key factor in developing new nanotechnology materials for different industries and applications, such as engineering and energy.

It was reported in several earlier works that mechanical alloying (MA) is an effective and easy process of obtaining nanosized microstructures with a higher content of reinforcement phases [13]. MA allows for controlled homogeneous distribution of the oxide particles in the grain boundaries; additionally, by applying a proper sintering technique, it is possible to obtain fully dense composites with enhanced mechanical properties [14]. The oxide dispersion-strengthened (ODS) steels are promising candidates as structural materials for application in advanced nuclear reactors because of their higher radiation resistance and enhanced mechanical properties [15]. The thermally stable oxide particles imbedded in the ODS steels provide greatly enhanced mechanical properties. It was reported that ODS composites with a high Cr content have good corrosion resistance in supercritical water reactors [16,17,18].

In our former work, our approach was to create a protective alumina layer in the grain boundaries, starting from embedded and stable ultra-fine alumina (Al_2_O_3_) powders instead of adding aluminium (Al) powders or Al-Fe coatings, as mentioned in most of the research conducted recently on this topic [19]. We assumed that the addition of alumina powders would provide a better Al_2_O_3_ protection layer for the steel composites due to their distribution along the grain boundaries instead of adding an insufficient amount of aluminium or Al-Fe powder to the protective coating formation. In addition, alumina is thermodynamically stable at the sintering temperature (900 °C) and allows for better control of sintering by avoiding the infiltration of aluminium into the 316L steel grains during the sintering process.

The CDS materials were produced by Spark Plasma Sintering (SPS). This method allows for rapid consolidation of powder materials into dense bulk specimens while simultaneously applying uniaxial pressure and pulsed electrical current in a vacuum or protective atmosphere. The importance lies in the fast (a few minutes) production and the variety of controlling the bulk material properties [20]. Earlier relevant studies related to the production of SPS samples are summarized in [1,21].

During diffusion bonding at an elevated temperature of around 60–70% of the melting temperature, a high pressure is applied for a longer period, typically 1–2 h. At the microscopic level, the peaks of the contact surfaces of the roughness profile first form a planarized interfacial boundary, whereas voids will be formed between the two contact surfaces. These voids disappear during the long bonding procedure through different surface mechanisms. The theoretical background is written by Hill and Wallach [22]. In the authors’ previous works [23,24,25], the bonded Gleeble experiments were studied and compared with the theoretical model for 316L/316L experiments. It is visible from earlier experiences [2] that the bonding quality is highly influenced by the selected bonding parameters (temperature, pressure, time) and depends significantly on the surface preparation (removal of contamination and oxides) [26]. For the diffusion bonding of sintered ODS steels (PM2000), a detailed theoretical model was developed [27,28].

In this work, the diffusion bonding of a newly developed 316L ceramic dispersion-strengthened (CDS) steel material to a standard steel was investigated.

## 2. Sample Preparations and Experimental Methods

In this work, the CDS materials were diffusion-bonded with 316L stainless steel rods with a Gleeble Physical Simulator (Dynamic Systems Inc., New York, NY, USA). Two types of specimens were prepared by powder metallurgy using the SPS technique based on earlier works; the manufacturing was the same as it is detailed in [29,30]. One type is made from 316L powder without additives, and the other was prepared with additional 1 wt% Al_2_O_3_ in the 316L powder matrix. Reference specimens without additives will be referred to as REF-316L; specimens with 1 wt% Al_2_O_3_ will be referred to as CDS-316L; and the wrought material from a cylinder is named 316L rod. In the present study, the results of the flexural strength, microhardness, and microstructural investigations of the welded specimens are presented.

**Figure 1:**
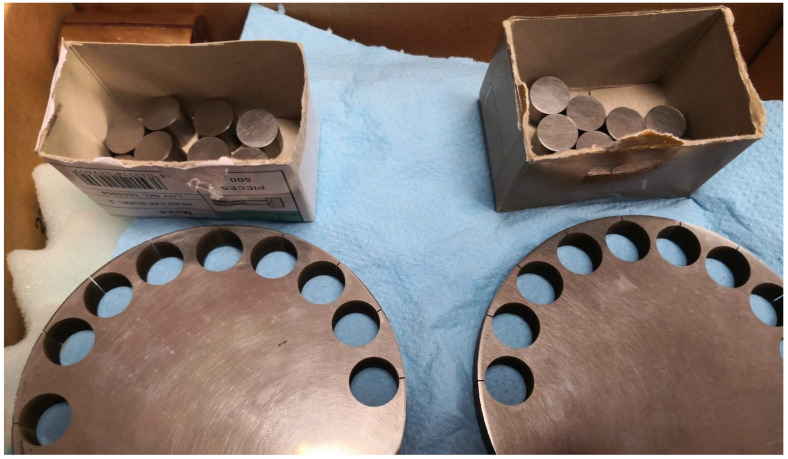
The original SPS sintered discs and D12.5 mm samples REF-316L (**left**), CDS-316L (**right**).

A Gleeble 3800 Physical Simulator at UNIDUNA [31,32] has been used to perform the bonding experiments on the REF-316L and CDS-316L composites. The original cylindrical CDS-316L or REF-316L discs sintered by SPS have dimensions of about 100 mm in diameter and 10 mm in height. After grinding and polishing the SPS discs, the cylindrical specimens were formed by wire-cut electrical discharge machining (Figure 1). The surface roughness characteristic values Ra, Rq, Rz were measured 2–3 times at all surfaces subjected to diffusion bonding by MITUTOYO SJ-301^®^ (Kawasaki, Japan) after surface polishing and before acid etching. The standard deviations for all measurements were ~1%.

The bonded surfaces, such as CDS-316L, REF-316L, and 316L rod, were polished with 600–1000/1200 grit abrasive paper under water. The surface roughness was measured on all surfaces subjected to diffusion bonding by MITUTOYO SJ-301^®^. The specimens were subsequently cleaned with acetone. To remove the Cr oxides from the surface, samples have been etched with a CITRANOX^®^ (Merck, Darmstadt, Germany) (acid solution (2–5% in deionized water) in an ultrasonic bath for 10 min at 35 °C [2]. However, the surface roughness has not had the largest impact on the bonding strength, as discussed in [26] but was set in the range of Rz = 0.5 to 2 µm. After the etching with an acid solution, the samples were cleaned subsequently with deionized water for 5 min in an ultrasonic bath. After drying, they were shipped under an inert Ar gas in a vacuum chamber, preventing reoxidation and contamination. Despite the cleaning process, the re-oxidation could not be fully avoided because of the preparation of the samples before the experiments. The specimens were exposed to air for 70–90 min (see Table 1). Presumably, in this case, the oxide layer was much thinner compared to what the original surface had.

### 2.1. Uniaxial Diffusion Bonding Experiments in the Gleeble 3800 Simulator

The uniaxial diffusion bonding process was carried out with 316L stainless steel rods, where the sintered composites were placed between the two 316L holding rods. A similar diffusion bonding setup on the Gleeble Physical Simulator was successfully applied in [33] to define the best bonding parameters of SS316L/CuCrZr with a nickel interlayer foil at various temperatures, where the loads were 650 °C, 850–1000 °C with a uniaxial pressure of 5–15 MPa and hold times of 15 min and 30 min. Uniaxial diffusion bonding was used to optimize bonding parameters such as 20 MPa, 40–120 min, and 1010–1050 °C in [23] between Eurofer 97 samples. Based on the mentioned experiments, relevant optimized parameters can be applied to different types of diffusion bonding techniques, like the HIP (hot isostatic pressure) technique.

**Figure 2:**
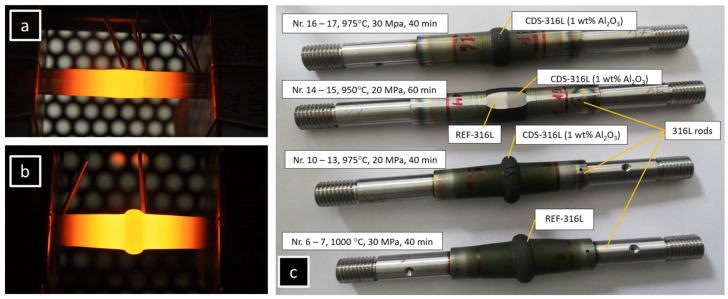
Diffusion bonding experiment, (**a**) Nr. 14–15: bonding of REF-316L and CDS-316L, (**b**) Nr. 6–7: bonding of REF-316L, (**c**) final bonded specimens.

The experiments and the bonded specimens are visible in Figure 2 and are detailed in Table 2. The Gleeble physical simulator was programmed, and the R-type thermocouples were spot-welded to the CDS/REF specimens and to the 316L-rods at the sides closed to the copper jaws. Afterwards, the full mount was assembled and installed into the vacuum chamber.

The specimens were heated up by resistance heating, and the temperature was controlled through an R-type thermocouple, which was spot welded as close as possible to the bonded region (CDS-316L or REF-316L). The axial pressure was controlled by the hydraulic system and measured by the load cell, where the pressure is set by the diameter and length and subsequently modified by the control system to keep the constant nominal pressure at 20 or 30 MPa. The nominal loads were set as detailed in Table 2.

**Figure 3:**
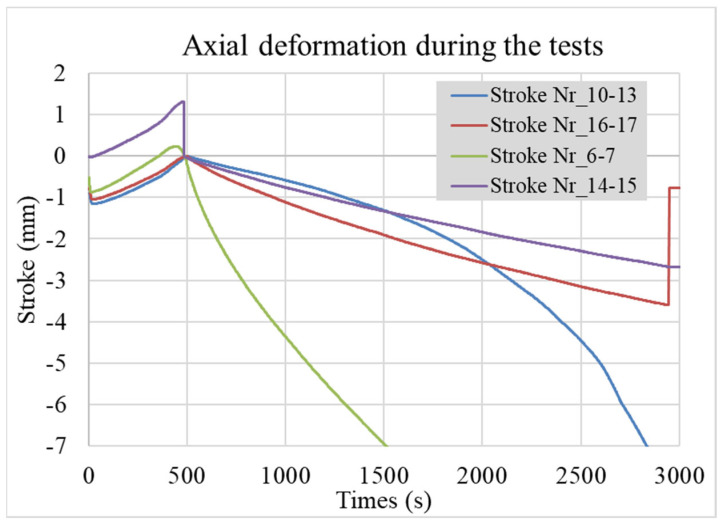
Axial deformation/stroke values of the specimens, shifted to “0 mm” at 500th sec.

**Figure 4:**
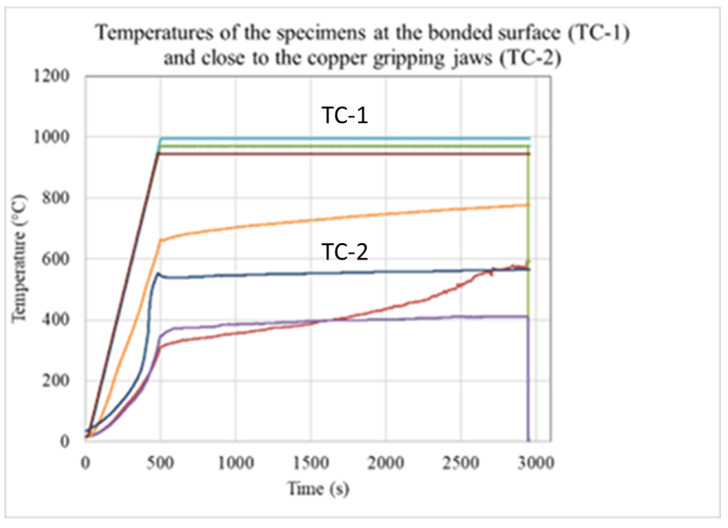
Measured temperatures at the bonded region (controlled) and closed to the gripping jaws during the bonding process.

The measured value of the sampling rate was 10 Hz. Beyond the controlled loads, the axial displacement (named stroke) and the Power Angle (PA) [%] fraction of applied power were recorded; the last is controlled by the Gleeble computer system automatically (Gleeble 3800). The typical measured values of the strokes are visible in Figure 3, and the measured temperatures during the bonding processes are visible in Figure 4.

As is visible, all bonding processes start with a 500 s period, where the heating-up takes place in a controlled way. The nominal bonding starts at the 500th second. The TC-1 temperatures follow the nominal control temperature at the bonded surface, but the side temperatures TC-2 close to the gripping jaws show again an increasing rate.

### 2.2. Preparation of the Mechanical and Microstructural Tests

**Figure 5:**
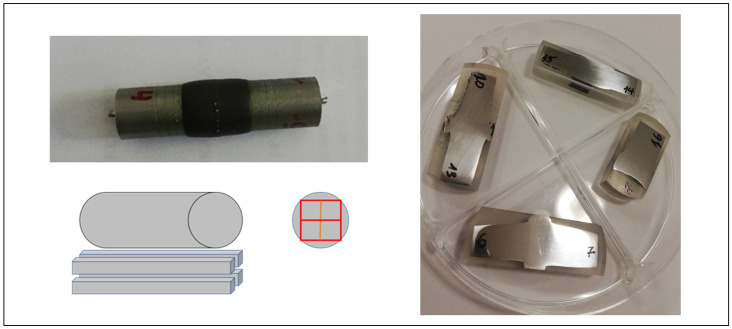
Preparation for samples for mechanical and microstructural investigations.

For micro-hardness and flexural strength (three-point bending) tests, four bars with dimensions of 4 × 4 mm × 40 mm have been prepared from each bonded specimen. The four bars were cut from the middle of the specimens, as shown in Figure 5. The samples have been polished using abrasion paper up to 1200 grade, where different zones across each specimen have been observed even with the naked eye.

Since during the cutting of the nr. 16–17, the specimen was broken, there were no further microstructural investigations on that sample; however, the other samples gave similar valuable results.

Scanning Electron Microscopy (SEM, Zeiss-SMT LEO 1540 XB, Oberkochen, Germany) was used for structural and morphological investigations of sintered samples. The elemental compositions of sintered samples were measured by Energy Dispersive Spectroscopy (EDS) installed on a SEM LEO microscope.

## 3. Results and Discussions

Following the previous studies [23], CDS-316L and REF-316L specimens were diffusion welded in the same way. According to Table 2, the first experiment was carried out at 1000 °C with a 30 MPa axial load. Since the elevated temperature and compression resulted in high deformation on the composite specimens, we decreased the temperatures for the following tests. Hereby, the other experiments were conducted at 975 °C and 950 °C and at 20 MPa axial compression. The lower loads (950 °C and 20 MPa) resulted in smaller deformations.

### 3.1. Evaluation of the Measured Values by Gleeble

**Figure 6:**
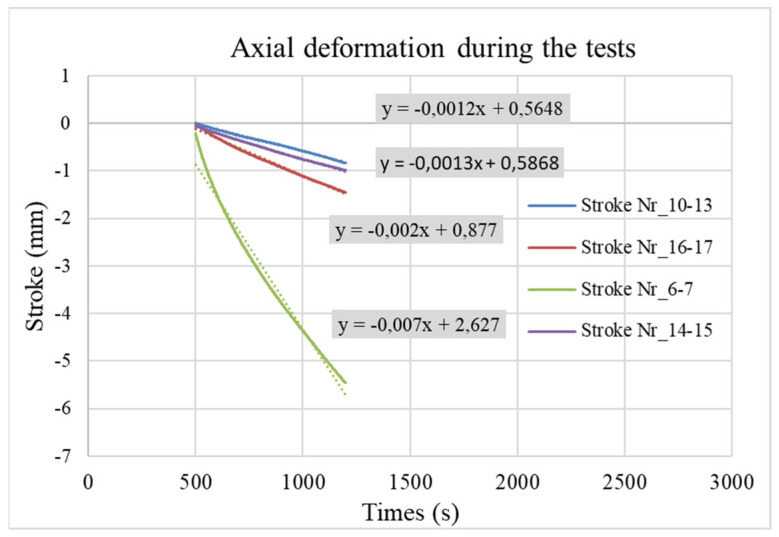
Axial deformation and stroke value during the relevant section in time.

The strokes (Figure 3) at the nominal starting point at 500 s (*y*-axis) were shifted to the “0 mm” point to be comparable. The rate of strokes in the different sintered specimens. Based on earlier measurements of the 316L rod’s axial deformation (stroke), the CDS-316L and REF-316 specimens could be estimated by subtracting the reference 316L rod’s stroke rate. The full nominal bonding time was 40 min between 500 and 2900 s. Since the specimens suffered a high degree of deformation, we considered the first half of the experiments: 500–1200 s. Results were linearized. See Figure 6. The rate of reference 316L rods and the CDS-316L and REF-316L specimen strokes are visible in Table 3 in the third and fourth column.

In all cases, the axial deformation (stroke) of the sintered samples was higher compared to the 316L rods during the tests.

**Figure 7:**
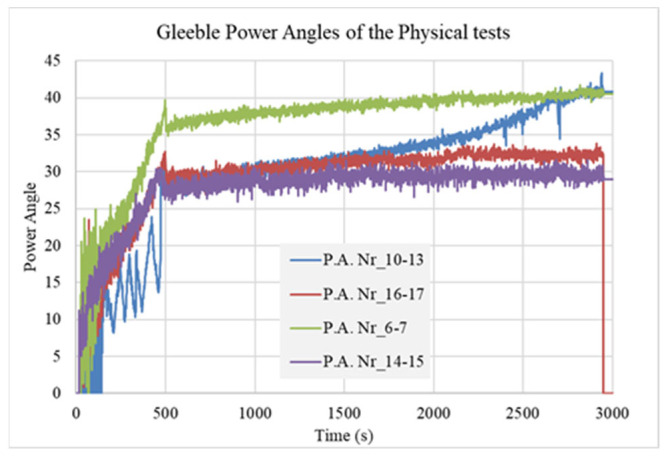
Measured values by the Gleeble system during the tests Power Angle.

However, the applied power could not be measured directly on Gleeble, but based on earlier numerical simulations [23], we expect 800–900 (A) current to pass through the specimens during the nominal bonding period. The Power Angles in the Gleeble control system also show the consumption of the full system with the ancillary units. It has no direct relation with the applied AC (50 Hz) current at Gleeble, but the curves show a very clear tendency in the ramp-up section and an increasing tendency as is expected during the boding process (Figure 7).

**Figure 8:**
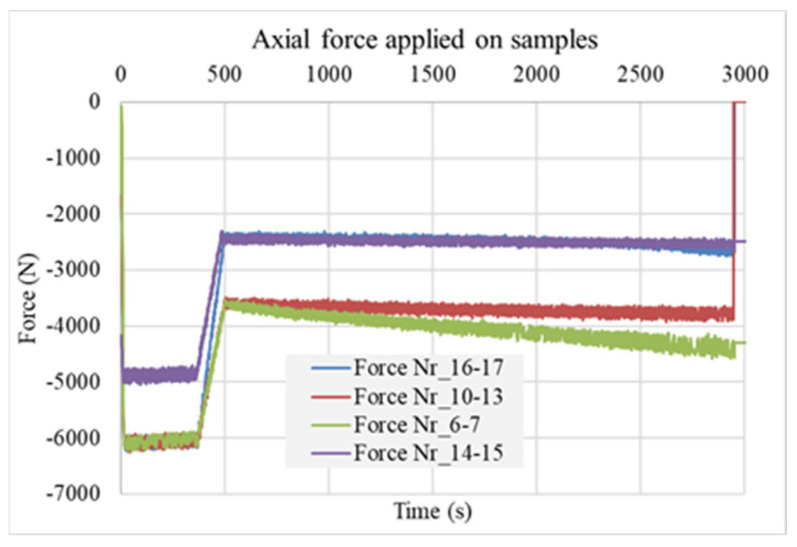
Measured force-axial force in the Gleeble system during the tests Power Angle.

The axial forces have an increasing tendency as well, since the Gleeble control system increases them to maintain the constant pressure with the increasing cross-section as in Figure 8.

We can conclude that the sintered specimens showed higher deformation compared to the 316L rods in all experiments (see Table 3). At 1000 °C (nr. 6–7), we obtained the highest axial stroke, where we set the highest 1000 °C temperature. For comparison purposes, nr. 16–17 and nr. 10–13 had the same sintered specimen and temperature but different pressures of 30 and 20 MPa. As was expected, nr. 10–13 had a lower stroke rate compared to nr. 16–17 until 1200–1500 s, but after that period, the stroke rate increased drastically. Since there was no explanation for that abnormality—that may have come from the control system—we used the more realistic values that were measured until 1500 s. Specimen nr. 14–15 had the lowest stroke rate at 950 °C and 20 MPa. Despite these lower temperatures and pressures, the samples still showed good flexural strength results, as summarized in the next chapter.

In our earlier studies, the strokes were modeled by the creep power law equation, with parameters set to the reference measurements [23,24,25]. Considering the studies on the effect of direct current on the steady-state creep of metals [34], we suppose that the high creep rate during the experiments is the outcome of the high temperature and the direct current running through the CDS specimens. For high-temperature creep rates of ODS austenitic steels, there are existing models from room temperature (RT) up to 800 °C [35]. In the future, an extensive study of creep under Joule heating of a CDS material around 1000 °C and the modeling of axisymmetric compression [36] could reveal this large deformation during the tests.

### 3.2. Mechanical Tests

#### Microhardness Results

**Figure 9:**
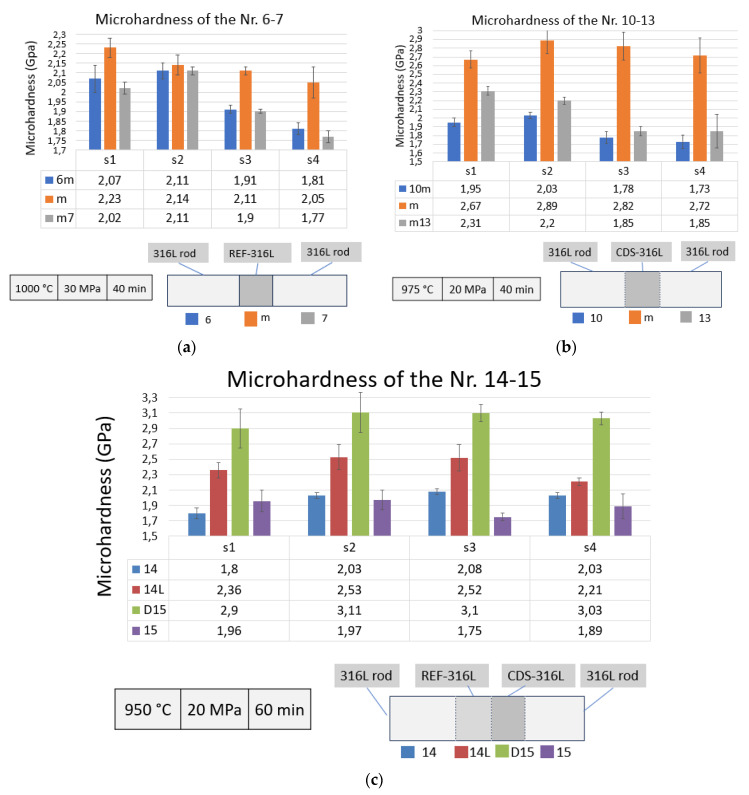
Microhardness results for the different zones of, (**a**) specimen nr. 6–7, (**b**) specimen nr. 10–13 (**c**) specimen nr. 14–15.

The mechanical properties of the bonded specimens were first analyzed with microhardness measurements, where an average of 12 measurements have been performed on each zone of all specimens. All results are summarized in Figure 9.

On specimens nr. 6–7, three different zones were observed. The zones are named 6 m, m, and 7 m, respectively, as represented in Figure 9a, where the hardest zone was REF-316L (reaching the 205–223 HV). However, the only difference between the 316L rods and the REF-316L zones is that the last is made by SPS powder metallurgy in front of the standard 316L rods. At nr. 10–13 specimen at lower deformation, under lower pressure (20 MPa) and temperature (975 °C), the CDS-316L composite in the middle shows much harder values (reaching 289 HV), see Figure 9b. As observed in an earlier study, the addition of alumina nanosized particles and the severe plastic deformation during the intensive attrition milling induced significant morphological changes and increased the microhardness of the CDS samples significantly [19]. The zones of the 316L rod at a lower deformation show similarly low values as the samples nr. 6–7.

The specimen nr. 14–15 in Figure 9c at a lower temperature (950 °C) compared to nr. 10–13 shows that the CDS-316L is harder (reaching 311 HV), and the REF-316L composite zone (14L) also shows harder values (reaching 250 HV). The 316L rods show similar lower values as the previous measurements.

We can observe that both REF-316L and CDS-316L composites have a relatively higher micro-hardness compared to the 316L rods, where at lower bonding temperatures (1000 °C → 975 °C, 950 °C), we obtained increasing hardness values.

We can also conclude that longer bonding time (t = 60 min) with lower pressure (P = 20 MPa) and temperature resulted in higher micro-hardness values reaching 311 HV comparing with a shorter bonding time (t = 40 min), with a higher pressure (P = 30 MPa) and temperature (T = 975 °C or 1000 °C). This is most likely related to the lower deformation of the steel grains.

### 3.3. Flexural Strength Results

**Figure 10:**
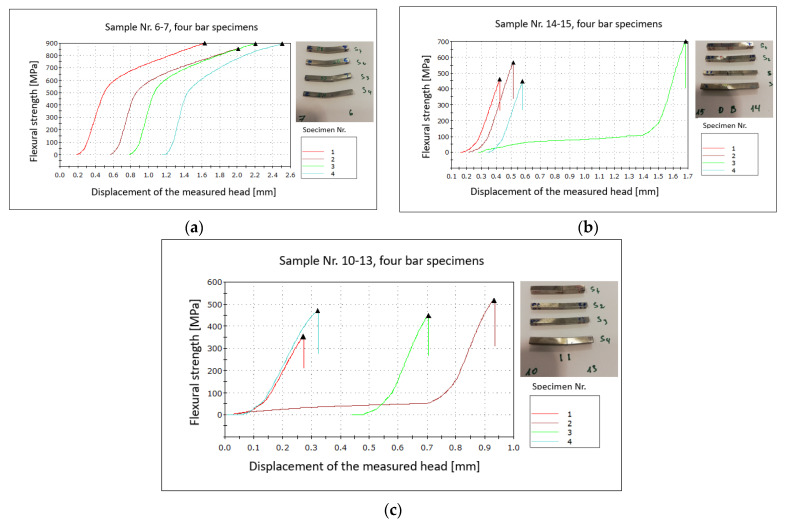
Flexural strength results (**a**) nr. 6–7, (**b**) nr. 14–15, (**c**) nr. 10–13.

**Figure 11:**
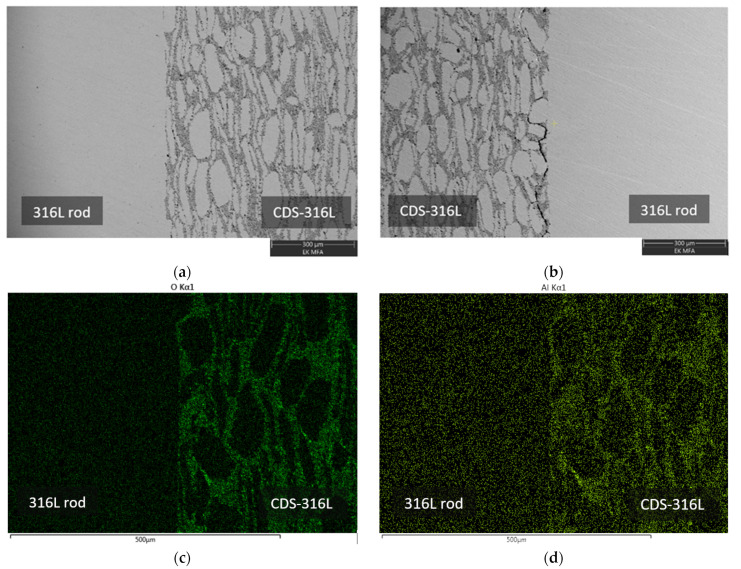
SEM and EDS investigations of specimen nr. 10–13 interface, (**a**) 316L-rod/CDS-316L interface, (**b**) CDS-316L/316L-rod interface showing the initial crack, (**c**,**d**) elemental mapping of the same interfaces.

The results of the three-point bending test are represented in Figure 10. The specimens nr. 6–7 were ductile, and fracture did not occur, as is visible on the Figure 10a insert. The diffusion-bonded REF-316L and CDS-316L samples (nr. 14–15) showed the highest flexural strength properties with an average of 543.75 MPa (Figure 10b), where the samples did not fracture at the interface but broke in Zone D due to their elongated grains in the parallel direction of the applied force and the presence of alumina particles in the grain boundaries (as shown in Figure 11).

The samples nr. 10–13 broke in the CDS-316 and the 316L-rod interface (Figure 10b) due to the presence of an initial crack along the diffusion-bonded interface as it was visible on the SEM images (as in Figure 11b).

The present experiments resulted in good bonding between the CDS-316L and the REF-316L composites, where during the three-point bending test, the crack was not at the interface, but at the harder CDS zones due to the elongated grains parallel to the applied force.

### 3.4. Microstructural Investigations

**Figure 12:**
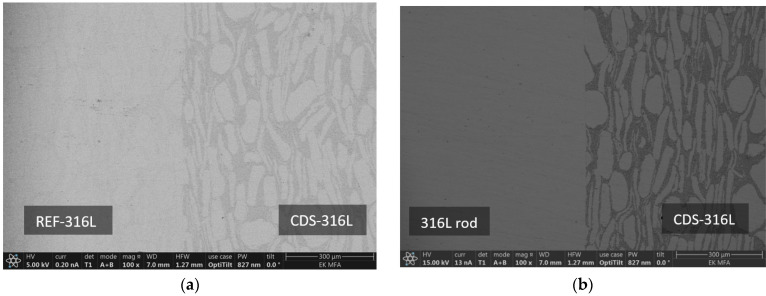

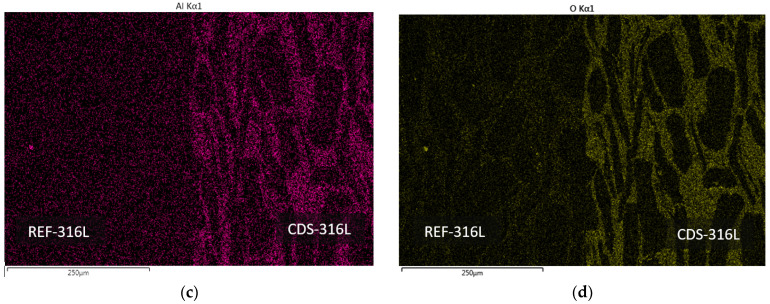
EDS and SEM Images of the bonded interface in nr. 14–15, (**a**) SEM image of the REF-316L and CDS-316L interfaces, (**b**) SEM image of the 316L rod and CDS-316L interfaces, (**c**,**d**) EDS elemental mapping of the same interfaces.

The bonded region of the specimens has been investigated. The SEM images of the CDS-316L region show a mixture of large grains and thin elongated grains surrounded by a darker phase; see Figure 11 and Figure 12. The EDS investigation revealed that the alumina particles are distributed in the dark zone. It is visible in Figure 11 and Figure 12c,d.

The interface between the CDS-316L and the 316L holding rod was well defined; no obvious diffusion zones have been observed within the investigated magnification. The SEM investigation of specimen nr. 10–13 between zone 316L rod and CDS-316L revealed the presence of an initial crack at the interface (see Figure 11b). The presence of the crack resulted in lower flexural strength by the three-point bending test, as shown in Figure 11b. The crack formation is possibly related to the residual stresses that originated during the cooling process.

The bonded interface of all samples has been observed; no initial cracks have been observed except for specimen nr. 10–13 (Figure 11b). Nr. 14–15 show the best flexural strength results, where the three-point bending test samples broke in Zone D (at CDS-316L). The samples were broken at the CDS zone, where the elongated grains are parallel to the broken surface.

Despite the crack formed at the Nr. 10–13 sample interface, all other investigated interfaces are well defined, and no diffusion zones have been observed within the investigated magnification.

**Figure 13:**
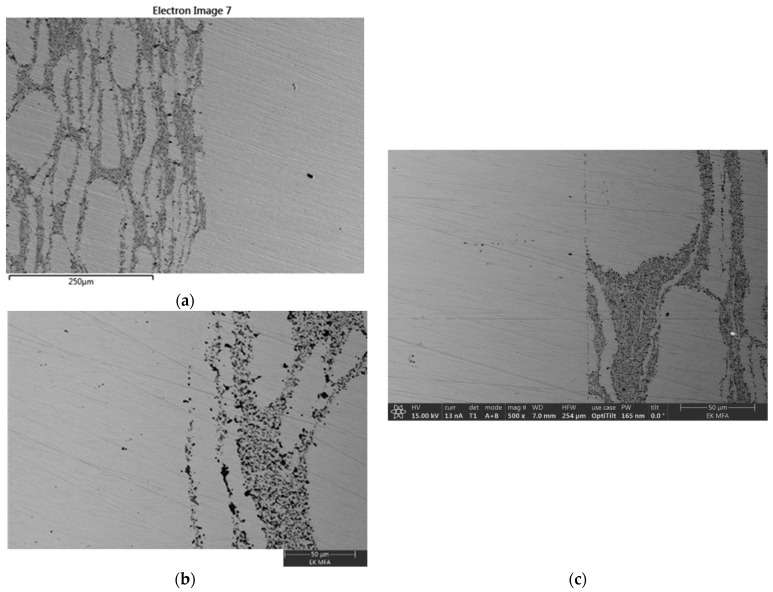
SEM Images of the bonded interface at Nr. 10-13 (**a**,**b**) shows the 316L-rod/CDS-316L, (**c**) 316L-rod/CDS-316L interface.

At higher magnification of the SEM microstructural images in Figure 11a and Figure 12b, we could also observe the joint surfaces. However, at higher magnification, the remaining void from the surface roughness’s could be seen in more detail, but at the present magnification, there were visible inclusions (Figure 13c) on the mating surfaces at the lower temperature and pressure combinations. Based on that, we could conclude that at higher temperatures and pressure combinations, the bonding shows better surfaces, as is expected from past results [2,22].

## 4. Conclusions

The SPS-sintered REF-316L and CDS-316L specimens were successfully diffusion-bonded with standard 316L stainless steel rods on a Gleeble 3800 physical simulator. The bonding parameters were within the temperature range of 950–1000 °C and axial mechanical pressures of 20–30 MPa, based on earlier works [23,24,25].

1. It was observed that the deformation of the CDS-316L specimens during the bonding process at high temperatures (>950 °C) was much higher compared to the standard 316L steel.
2. The CDS-316L shows higher hardness values due to the alumina content in the specimen.
3. The results of the three-point bending test were between 440 and 550 MPa. The flexural strength of the Nr. 14–15 samples (316L rod/CDS-316L/REF-316L/316L rod) was the highest, with an average of 543.75 MPa.
4. At the bonding interface, no diffusion zones have been observed within the investigated magnification for all composites, and the interfaces between the different zones were well defined.
5. In the case of the Nr. 10–13 sample (316L rod/CDS-316L/316L rod), where an initial crack was observed by SEM, the flexural strength was accordingly lower, where the samples showed fracture at the interfaces.
6. The CDS-316L samples consist of a mixture of large and elongated grains surrounded by a darker area rich in oxygen and aluminium, showing the alumina particles are distributed in the darker areas of the investigated samples.
7. Longer bonding times with lower pressure and temperature resulted in higher micro-hardness values.

The future work on this topic will focus on the modeling of specimens’ deformation during the bonding process in the Gleeble 3800 physical simulator. Furthermore, the optimization of the bonding of the CDS-316L composites at the proper loads, aiming for the highest possible mechanical properties with a good joint, will provide more results in future works.

## Figures and Tables

**Table 1:** The size and surface roughness values of the specimens were used in the bonding experiments.

Nr.	Height (mm)	D (mm)	Side A			Side B			On Air
			Ra (μm)	Rz (μm)	Rq (μm)	Ra (μm)	Rz (μm)	Rq (μm)	
Nr. 6–7 (REF)	10.25	12.43	0.09	0.80	0.11	0.10	0.85	0.13	70 min
Nr. 10–13 (CDS)	8.96	12.43	0.11	1.02	0.14	0.10	0.78	0.12	90 min
Nr. 14–15 (CDS)	n. a. ~ 9	12.45	0.09	2.88	0.21	0.10	0.97	0.12	90 min
Nr. 14–15 (REF)	n. a. ~ 10.25	12.45	0.23	1.74	0.35	0.11	0.94	0.14	

**Table 2:** Diffusion bonding parameters and the length change.

Nr.	Sample Layout	T [°C]	P [MPa]	t [min]	Length before and after [mm]
Nr. 6–7	316L rod/REF-316L/316L rod	1000	30	40	71/57.03
Nr. 10–13	316L rod/CDS-316L/316L rod	975	20	40	69.04/60.84
Nr. 16–17	316L rod/CDS-316L/316L rod	975	30	40	68.95/65.27
Nr. 14–15	316L rod/CDS-316L/REF-316L/316L rod	950	20	60	80/76.5

**Table 3:** Rate of strokes of the 316L rods and the composite specimens in mm/min.

Exp.	Rate of Strokes of the Full Specimens (mm/min)	Rate of Strokes of Reference 316L Rods (mm/min)	Derived Rate of Strokes for the CDS, REF Specimens (mm/min)	
Nr. 10–13	−0.069	−0.003	−0.072	CDS-316L
Nr. 16–17	−0.118	−0.0126	−0.1306	CDS-316L
Nr. 6–7	−0.414	−0.0953	−0.5093	REF-316L
Nr. 14–15	−0.08	−0.002	−0.082	CDS-316L + REF-316L

## Data Availability

Data are contained within the article.
